# Volcanic glass geochemical fingerprints of the Black Sea tephra layers in the last ~60 kyr

**DOI:** 10.1038/s41597-026-07504-9

**Published:** 2026-06-01

**Authors:** Ivan Sunyé-Puchol, Xavier Bolós, Rengin Özsoy-Ünal, Victoria C. Smith, Lorenzo Tavazzani, Manuela Nazzari, Victoria Cullen, Olivier Bachmann, Piergiorgio Scarlato, Silvio Mollo

**Affiliations:** 1 Department of Earth Sciences, Sapienza University of Rome, Piazzale Aldo Moro 5, Roma, 00185 Italy; 2 Geosciences Barcelona (GEO3BCN), CSIC, 08028 Barcelona, Catalonia Spain; 3 School of Archaeology, University of Oxford, 1 South Parks Road, Oxford, OX1 3TG United Kingdom; 4 Institute of Geochemistry and Petrology, ETH Zürich, Clausiusstrasse 25, Zurich, Switzerland; 5 Istituto Nazionale di Geofisica e Vulcanologia (INGV), Sezione di Roma1, Rome, Italy; 6 Department of Biochemistry, University of Oxford, Oxford, United Kingdom

## Abstract

Tephra layers preserved in marine sedimentary archives constitute key isochronous markers for reconstructing volcanic activity and regional stratigraphic frameworks. Here we present a comprehensive geochemical dataset of volcanic glass from tephra layers identified in sediment core M72/5-25-GC1 recovered from the southwestern Black Sea. The dataset includes major and trace element compositions of volcanic glass shards from 19 tephra layers deposited in the last ~60 kyr, preserved as both visible tephra and cryptotephra horizons. Glass shards were analyzed using electron probe micro-analyzer and sector-field laser ablation inductively coupled plasma mass spectrometry, following rigorous analytical protocols and quality control procedures. The dataset spans a wide compositional range, reflecting inputs from multiple volcanic regions, including the Central Anatolian Volcanic Province, the Aegean arc, and Italian volcanic districts. This dataset provides a robust geochemical framework for tephra fingerprinting and correlation across the Black Sea and surrounding areas, and represents a valuable resource for tephrochronological applications, paleoenvironmental reconstructions, and future comparative or data-driven studies.

## Background & Summary

**Fig. 1:**
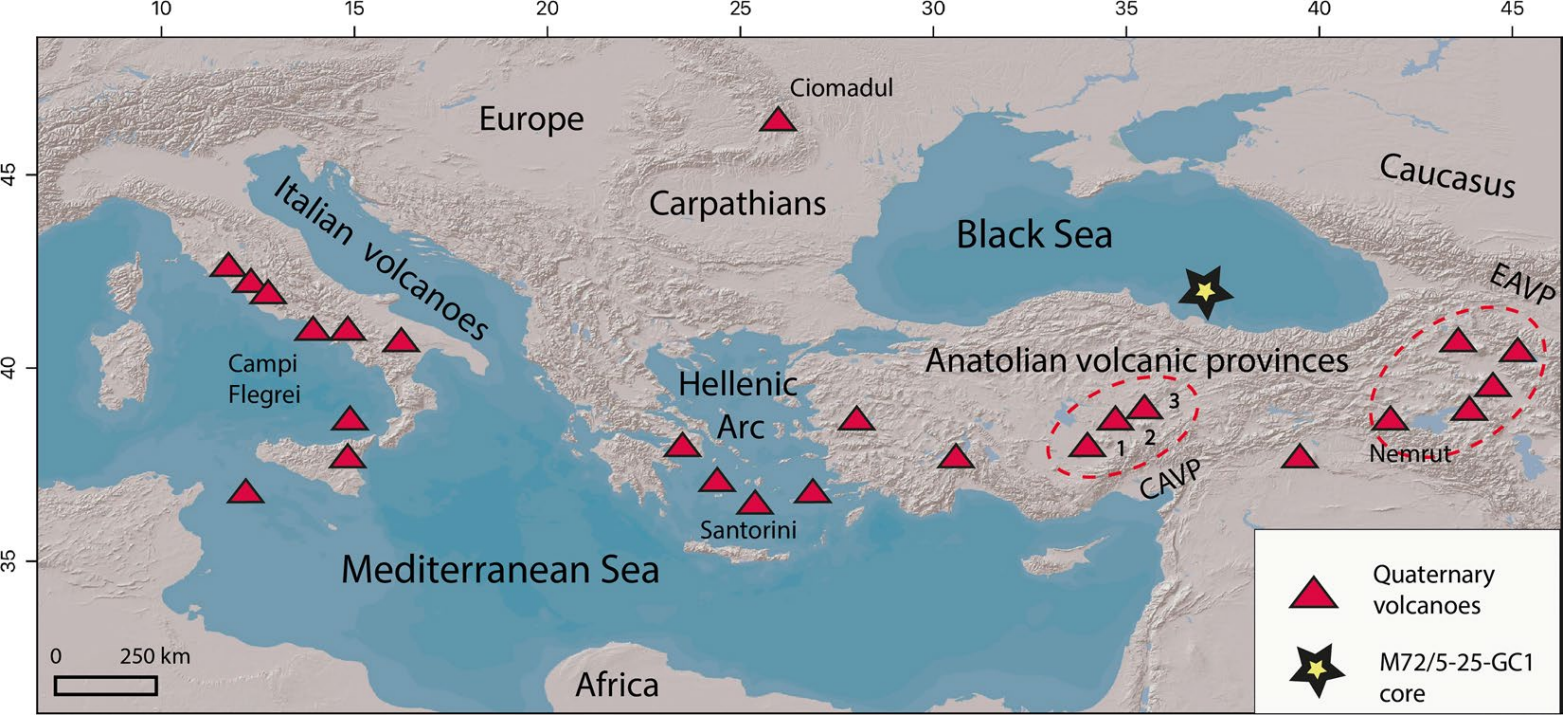
Location of sediment core M72/5–25-GC1 and regional volcanic setting. Overview map of eastern Europe, the Mediterranean region, and the Black Sea showing the principal volcanic provinces contributing tephra to the basin, including Italian, Aegean, and Anatolian volcanic regions. CAVP: Central Anatolian Volcanic Province, EAVP: Eastern Anatolian Volcanic Province, 1 = Hasandağ, 2 = Acigöl, 3 = Erciyes.

The tephra layers dispersed and deposited over wide areas during explosive volcanic eruptions constitute unique time-synchronous stratigraphic markers and are widely used in tephrochronology, paleoenvironmental research, and volcanological studies^1,2^. Marine sedimentary archives are particularly valuable in this context because they provide continuous and regionally extensive records of tephra fallout that preserve both visible tephra layers and cryptotephra horizons, which are not detectable by visual inspection alone^1,2^. Among these archives, the Black Sea represents a key repository of distal tephra deposition, as it receives volcanic ash from multiple surrounding volcanic regions in eastern Europe and the Mediterranean, including the Aegean arc, the Anatolian volcanic provinces, and Italian volcanic districts (Fig. 1)^3–5^. Owing to its semi-enclosed nature and relatively high sedimentation rates^6^, the Black Sea basin preserves detailed and stratigraphically coherent records of distal tephra layers derived from large explosive eruptions.

The potential of Black Sea sediments for distal tephrochronology was first demonstrated by Cullen *et al*.^3^, who documented both visible tephra layers and cryptotephra horizons in core M72/5–25-GC1. The marine record was recovered from the southeastern Black Sea continental slope during a RV *Meteor* cruise in 2007^6^. The core was retrieved at a water depth of approximately 418 m from a distal slope setting characterized by fine-grained sedimentation and relatively high and continuous sediment accumulation during the late Pleistocene. This depositional environment is well suited to preserving long, stratigraphically coherent sedimentary records with high potential for recording distal tephra fallout^6^. The age models proposed by Nowaczyk *et al*.^6,7^, and in particular the high-resolution paleointensity stack of Nowaczyk *et al*.^7^, provide the chronological constraints adopted by Cullen *et al*.^3^ for reconstructing the tephrostratigraphy of the Black Sea core (BSC) M72/5–25-GC1, giving an age of ~60–63 ka to the base of this sedimentary record.

Within core M72/5–25-GC1, Cullen *et al*.^3^ identified a substantial dataset of (crypto-)tephra layers (Table 1), preserved as discrete stratigraphic horizons ranging from millimeter-thick visible ash layers (i.e., BSC-721) to cryptotephra deposits characterized by variable, yet commonly well-defined peaks in volcanic glass shard concentrations (i.e., Group A1, A2, B and C). These glass shards are typically in the few-tens-of-micrometers size range. Based primarily on major element compositions of BSC volcanic glass shards, and comparing it to published proximal glass chemistry data (e.g.^8–10^), Cullen *et al*.^3^ proposed correlations for several key horizons, including: (1) the well-known regional marker Y5 tephra associated with the ~39 ka BP Campanian Ignimbrite eruption (Campi Flegrei caldera, Italy)^11^; (2) the Z2 tephra associated with the ~3.5 ka BP Minoan eruption (Santorini caldera, Greece)^12,13^; and (3) late Pleistocene to Holocene eruptions sourced from Erciyes and Acigöl volcanoes^14^, located in the Central Anatolian Volcanic Province (CAVP; Fig. 1). However, owing to the small size of distal glass shards and analytical limitations at the time, trace element data were available for only a limited subset of tephra layers, and several of the ash horizons could not be confidently attributed to specific eruptions or volcanic sources.

**Table 1:** (Crypto-) Tephra layers in core M72/5-25-GC1 and correlations proposed by Cullen *et al*.^3^.

Sample code and glass shards (g^-1^)	Composition	Source volcano	Eruption	Eruption age	References	Dating Method
*Group C*						
BSC-022 (2683)	CAR	Santorini	Minoan (Z2)	3.68–3.58 ka	^12,13^	^14^C
BSC-079 (53000)	CAR	Erciyes	Karagüllü or Perikartin	6.4–10.5 ka/ 6.3 -8.9 ka	^31–33^	^36^Cl, (U-Th)/He, ^14^C
BSC-154 (320)	CAR	Acıgöl? CAVP?	Unknown	~14.6 ka	^6,7^	INT
BSC-158 (340)	CAR	Acıgöl? CAVP?	Unknown	~14.6 ka	^6,7^	INT
*Group B*						
BSC-394(306425)	CAR	Acıgöl	Güneydağ or Korudağ	23.8 ± 2.1 or 24.9 ± 2.1 ka	^14^	(U-Th)/He
BSC-411 (22867)	CAR	Acıgöl	Güneydağ or Korudağ	23.8 ± 2.1 or 24.9 ± 2.1 ka	^14^	(U-Th)/He
*Group A2*						
BSC-651 (180)	CAR/PR	CAVP? Nemrut?	Unknown	< 34.5 ± 0.65 ka (M.L. Excursion)	^34^	^40^Ar/^39^Ar and K/Ar
BSC-660 (50)	CAR	Unknown	Unknown	~34.4 ka	^6,7^	INT
BSC-674 (40)	CAR	Unknown	Unknown	~35.6 ka	^6,7^	INT
BSC-683 (67)	CAR	Unknown	Unknown	~36.4 ka	^6,7^	INT
BSC-694 (81)	CAR	Unknown	Unknown	~37.5 ka	^6,7^	INT
BSC-698 (38)	CAR	Unknown	Unknown	~37.8 ka	^6,7^	INT
BSC-705 (55)	CAR	Unknown	Unknown	~38.4 ka	^6,7^	INT
*Visible layer*						
BSC-721 (66467)	PT	Campi Flegrei	CI (Y-5)	39.28 ± 0.11 ka	^11^	^40^Ar/^39^Ar
*Group A1*						
BSC-729 (106)	CAR	Unknown	Unknown	~40 ka	^6,7^	INT
BSC-740 (78)	CAR	Unknown	Unknown	~40.7 ± 0.95 ka (L. Excursion)	^35^	^40^Ar/^39^Ar
BSC-752 (71)	CAR	Unknown	Unknown	~42.1 ka	^6,7^	INT
BSC-778 (150)	CAR	Unknown	Unknown	~44.5 ka	^6,7^	INT
BSC-807 (38)	CAR	Unknown	Unknown	~48.3 ka	^6,7^	INT
Base of the core				~60-63 ka	^6,7^	

BSC, Black Sea Core; CAR, Calc-alkaline rhyolite; CAVP, Central Anatolian Volcanic Province; PR, Peralkaline rhyolite; PT, Peralkaline trachyte, CI, Campanian Ignimbrite. Dating methods: ^14^C, radiocarbon dating; U-Th/He, uranium-thorium/helium dating; ^40^Ar/^39^Ar, argon-argon dating; K-Ar, potassium-argon dating; INT, interpolation within the published core age model.

The integration of detailed stratigraphic control with robust geochemical fingerprinting is essential for achieving reliable correlations of tephra layers across marine and terrestrial archives^15,16^. Successful distal tephra correlations critically depend on the availability of high-quality, well-documented geochemical datasets of volcanic glass compositions^17^. However, at the time, such datasets were largely restricted to major element compositions, while openly accessible trace element datasets for Black Sea tephras remained scarce, thereby limiting their systematic reuse and integration into broader tephrochronological frameworks.

Here we further develop the tephrostratigraphic framework of Cullen *et al*.^3^ by presenting a comprehensive geochemical dataset of volcanic glass shards for 19 (crypto-)tephra layers identified in core M72/5–25-GC1 (Table 1). By incorporating precise trace element compositions, made possible by recent advances in laser ablation instrumentation, this dataset provides new geochemical constraints. Analytical protocols were designed to ensure data quality and reproducibility through extensive method validation using international reference glasses^18^. The resulting dataset resolves both intra- and inter-layer variability in major and trace element compositions and establishes a solid geochemical framework for tephra fingerprinting. These new geochemical data are intended to support future correlations between Black Sea tephra layers and both proximal and distal volcanic deposits, also facilitating the transfer of isochronous markers across marine and terrestrial archives. The documented geochemical fingerprints are particularly relevant to volcanism in the CAVP, while also encompassing tephra sourced from other volcanic regions, underscoring the broader applicability of the dataset. Beyond tephrochronological applications, the data are suitable for reuse in paleoenvironmental reconstructions, comparative geochemical studies, and emerging data-driven approaches, including statistical and machine-learning-based correlation methods.

## Methods

### Electron microprobe analyses

Major oxide analyses of glass shards were collected using a JEOL JXA-8200 electron probe micro-analyzer (EPMA) equipped with five wavelength dispersive spectrometers (WDS) and installed at the Laboratory of Experimental Volcanology and Geophysics (HP-HT Lab) of the Istituto Nazionale di Geofisica e Vulcanologia (INGV) in Rome (Italy). We selected the ten canonical major oxide components commonly used to quantify glass compositions in volcanology and petrology: SiO_2_, TiO_2_, Al_2_O_3_, FeO, MnO, MgO, CaO, Na_2_O, K_2_O, and P_2_O_5_, with all iron expressed as FeO. Prior to major oxide measurements, glass shards were examined using backscattered-electron (BSE) imaging to identify compositionally homogeneous regions suitable for quantitative analysis. Only areas showing uniform grey-scale contrast in BSE mode, indicative of constant mean atomic number, were selected. Electron microprobe analyses were performed under high vacuum conditions with an accelerating voltage of 15 kV, an electron beam current of 7.5 nA, and a defocused beam diameter of 10 μm. Elemental counting times were 10 s on the peak and 5 s on each of the two background positions. The ZAF (Z: atomic number; A: absorption; F: fluorescence) procedure was applied to correct for inter-elemental effects. Calibration utilized a range of standards from Micro-Analysis Consultants Ltd (MAC; https://www.macstandards.co.uk): albite (Si-PET, Al-TAP, Na-TAP), forsterite (Mg- TAP), augite (Fe-LIF), apatite (Ca-PET and P-TAP), orthoclase (K-PET), rutile (Ti-PET), and rhodonite (Mn-LIF). Sodium and potassium were analyzed first to prevent alkali migration effects. MAC-Augite (brass block no. 6555) and MAC-Obsidian (brass block no. 11763) were used as quality monitors and for the calculation of accuracy and precision.

### Laser ablation analyses

Trace element analyses of glass shards were performed using a 193 nm excimer laser (RESOlution-LR; Applied Spectra) coupled with a second-generation two-volume constant geometry ablation cell (S-155) and a high-sensitivity, sector-field laser ablation-inductively coupled plasma mass spectrometer (SF-LA-ICP-MS; Thermo-Element XR) at the Institute of Geochemistry and Petrology of the ETH Zürich (Switzerland). We selected trace elements representative of key geochemical groups in glass geochemistry: large ion lithophile elements (LILE; e.g., Rb, Sr, Ba), high field strength elements (HFSE; e.g., Ti, Zr, Ta), transition metals (TM; e.g., Cr, Ni, V), rare earth elements (REE; La-Lu series plus Y, where yttrium is treated as a pseudo-lanthanide), and actinide elements (Act; Th, U). Points with a nominal diameter of 20 μm were set on glass shards previously analyzed by EPMA and ablated for 30 s with a pulse rate of 5 Hz and an energy density of ca. 3.5 J/cm^3^. Synthetic glass NIST SRM610^18^ was used as primary reference material for normalization and instrumental drift correction. To evaluate the analytical accuracy and reproducibility, the synthetic glasses BCR-2G^19^ and GSD-1G^20^ from the United States Geological Survey (USGS) were used as secondary reference materials. The CaO content of glass shards, determined by EPMA, was used as internal standard to convert raw counts to absolute contents. CaO exhibits low intrinsic variability across the dataset, with a coefficient of variation generally less than 10%. Raw data were processed using Iolite 4 (Iolite Software Ltd.)^21,22^. Both single-spot and time-resolved signals were carefully inspected for instability, anomalous spikes, and systematic trends indicative of contamination by microlites, interactions with microcracks/vesicles, or surface impurities on the glass shards. This pre-screening allowed the identification and exclusion of non-glass phases, by minimizing analytical artefacts related to mixed-phase ablation, surface contamination, or incomplete ablation of the glass matrix.

## Data Records

The dataset is available at the Figshare data repository under the title “Major and trace element glass geochemistry, validation model and microscopy images of Black Sea marine tephra layers”^23^ (10.6084/m9.figshare.31223815). The repository page provides the dataset metadata, file descriptions, DOI, license information, and direct access to the deposited data files.

The repository includes spreadsheet files containing the major oxide and trace element compositions of individual volcanic glass shards analyzed from tephra layers in core M72/5-25-GC1. The EPMA dataset reports sample code, tephra layer, analytical point identifier, original analytical totals, and major oxide concentrations for SiO₂, TiO₂, Al₂O₃, FeO, MnO, MgO, CaO, Na₂O, K₂O, and P₂O₅, with all iron expressed as FeO. Major oxide compositions are reported both as original analytical totals and as values normalized to 100 wt.% to facilitate direct compositional comparison.

The SF-LA-ICP-MS dataset reports trace element concentrations for individual glass shards, together with the standard error of the mean (SE), calculated from time-resolved signal integration, and limits of detection (LOD), defined as three times the standard deviation of the background signal following data reduction in Iolite 4. The repository also includes analytical quality-control files for the reference materials used to evaluate accuracy and precision, including MAC-Obsidian and MAC-Augite for EPMA analyses, and BCR-2G and GSD-1G for SF-LA-ICP-MS analyses.

Representative microscopy images of selected tephra samples and volcanic glass shards are also provided in the repository, including back-scattered electron images and petrographic microscope images. These images document shard morphology, vesicularity, preservation state, textural features, and analytical target selection, thereby complementing the quantitative geochemical data.

The repository further includes the spreadsheet used to support the statistically based petrochemical validation described in the Technical Validation section. This file contains the Zr-Hf reference compilation, the iteratively reweighted least-squares regression workflow, and the calculations used to compare the Black Sea glass shard compositions with the expected geochemical variability of rhyolitic compositions. This file is provided as supporting validation material and does not constitute an additional primary geochemical dataset.

## Data Overview

**Fig. 2:**
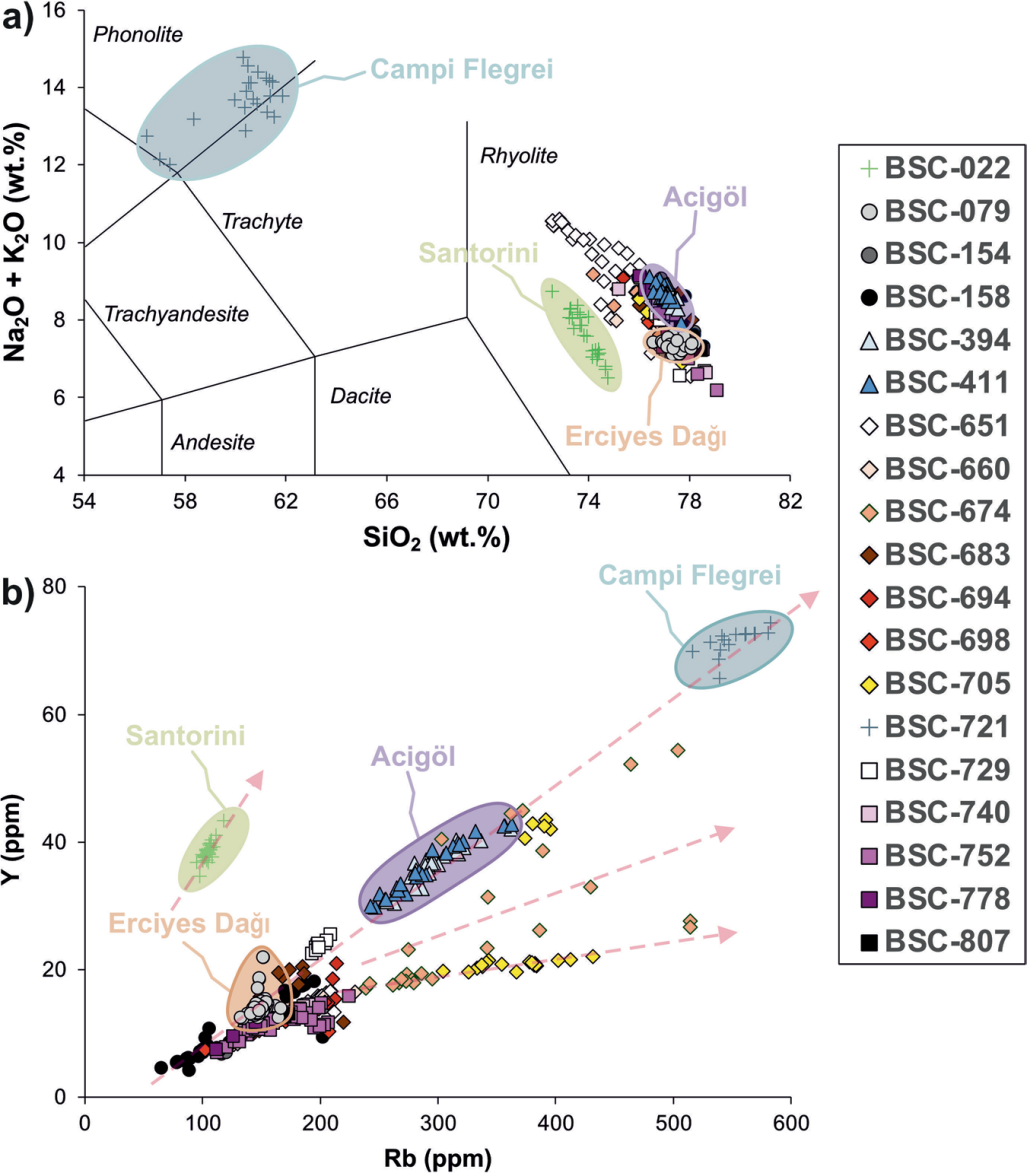
(**a**) Total alkali vs. silica (TAS) diagram showing the major element compositions of volcanic glass shards from 19 tephra layers identified in core M72/5-25-GC1. All samples plot within the rhyolite field, apart from BSC-721, which lies along the phonolite–trachyte boundary. (**b**) Rb vs. Y diagram illustrates distinct geochemical trends associated with different volcanic sources, including Campi Flegrei, Santorini, Acıgöl caldera, and Erciyes Dağı. Together, these diagrams underscore the effectiveness of combining major and trace element data to discriminate tephra provenance in the Black Sea region.

The dataset contains the major oxide and trace element compositions of glass shards from 19 different tephra layers (visible and cryptotephra) in core M72/5-25-GC1. According to the TAS classification diagram (Total Alkali vs. Silica)^24^ displayed in Fig. 2a, all samples plot within the rhyolite field (~73-79 wt.% SiO_2_ and ~6-10 wt.% Na_2_O + K_2_O), with the exception of sample BSC-721 from Campi Flegrei^3^, which lies along the boundary between the phonolite and trachyte fields (~57-61 wt.% SiO_2_ and ~12-15 wt.% Na_2_O + K_2_O). In addition, sample BSC-022 from Santorini caldera^3^ defines a distinct rhyolitic compositional group relative to the other samples, characterized by comparatively lower silica contents with respect to total alkalis (~73-75 wt.% SiO_2_ and ~7-10 wt.% Na_2_O + K_2_O). Notably, within the compositional variability of rhyolitic glass shards, samples BSC-394 and BSC-411 (~76-78 wt.% SiO_2_ and ~8-9 wt.% Na_2_O + K_2_O) cluster within a narrow compositional field previously attributed to Acigöl caldera eruptions from the CAVP^3^. A similarly restricted compositional range characterizes sample BSC-079 (~77-78 wt.% SiO_2_ and ~7-8 wt.% Na_2_O + K_2_O), representing glass shards from Erciyes volcano^3^ located in the CAVP.

In terms of incompatible trace element compositions, the Rb-Y diagram displayed in Fig. 2b highlights distinct data arrays that define separate evolutionary trajectories, along which both rubidium and yttrium increase systematically (~65-583 ppm Rb and ~4-74 ppm Y). Specifically, sample BSC-721 is characterized by markedly elevated trace element concentrations (~517-583 ppm Rb and ~66-74 ppm Y), reflecting the strongly evolved potassic to ultrapotassic alkaline magmatism of Campi Flegrei associated with an extensional back-arc setting. A comparable observation applies to the distinct geochemical array defined by sample BSC-022 (~95-118 ppm Rb and ~35-43 ppm Y), which represents calc-alkaline to high-K calc-alkaline arc magmatism at Santorini, driven by subduction along the Hellenic margin. Samples BSC-394 and BSC-411 from Acigöl are clearly distinguished by their trace element systematics (~247-356 ppm Rb and ~30-44 ppm Y), whereas sample BSC-079 from Erciyes clusters at lower trace element concentrations (~133-167 ppm Rb and ~13-22 ppm Y).

**Fig. 3:**
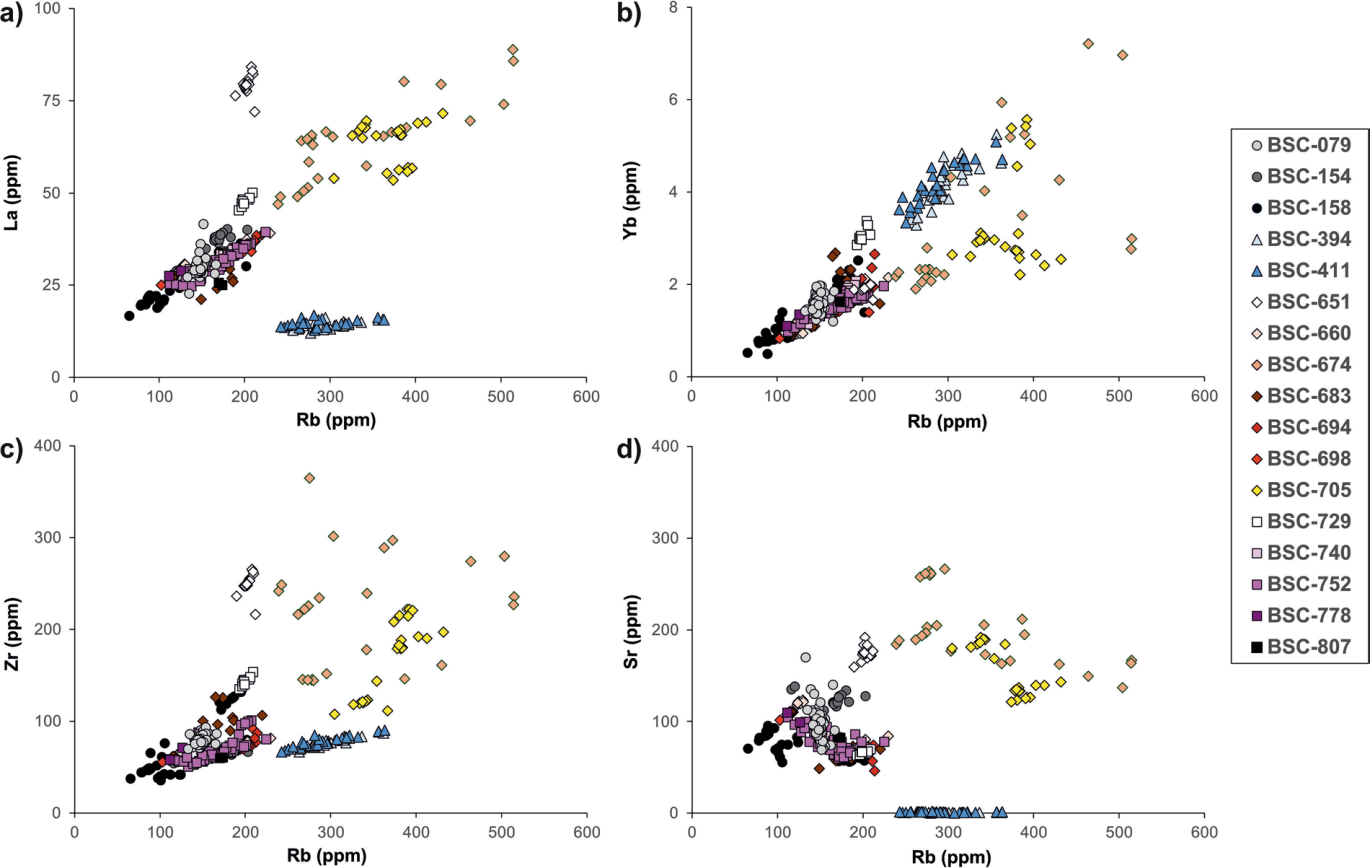
Representative trace elements plotted against Rb used as a differentiation index, including (**a**) La, (**b**) Yb, (**c**) Zr, and (**d**) Sr. Distinct compositional arrays reflect different magmatic affinities of the volcanic sources influencing the Black Sea region, particularly those from the Central Anatolian Volcanic Province. To enhance visualization of Anatolian compositional arrays, trace element data from Campi Flegrei and Santorini (i.e., BSC-022 and BSC-721) are excluded from selected panels.

To facilitate data visualization and comparison, the exotic samples BSC-022 (Santorini) and BSC-721 (Campi Flegrei) are excluded from the trace element plots illustrated in Fig. 3. The individual panels show representative trace elements, including light (La; Fig. 3a) and heavy (Yb; Fig. 3b) REE, HFSE (Zr; Fig. 3c) and LILE (Sr; Fig. 3d), all plotted against rubidium concentration, which is used as a differentiation index. Most samples define overlapping compositional fields, forming a broad compositional group with Rb of ~65-225 ppm, La of ~17-50 ppm, Yb of ~0.5-3.3 ppm, Zr of ~38-153 ppm, and Sr of ~46-170 ppm. In contrast, samples BSC-674 and BSC-705 extend to markedly higher Rb concentrations, reaching up to ~515 ppm, and exhibit a wider dispersion in trace element abundances, with La ranging from ~47-89 ppm, Yb from ~1.9-7.2 ppm, Zr from ~107-365 ppm, and Sr from ~123-266 ppm. Samples BSC-394 and BSC-411 define a distinct geochemical array, displaying extremely low La (~12-16 ppm) and Sr (~0.6-1.6 ppm) concentrations and moderately low Zr contents (~67-90 ppm). Additionally, sample BSC-651 is strongly enriched in La (~72-84 ppm) and moderately enriched in Zr (~217-261 ppm) and Sr (~159-192 ppm) relative to Rb concentrations (~189-208 ppm). As noted by Cullen *et al*.^3^, sample BSC-651 may not derive from a calc-alkaline volcanic source but instead reflects a peralkaline affinity within the Eastern Anatolian Volcanic Province (EAVP; Fig. 1), such as Nemrut volcano (Table 1). For the remaining tephra layers previously classified as of unknown origin by the authors (Table 1), the combined major and trace element compositions presented here show no geochemical affinities with Campi Flegrei, Santorini, or peralkaline sources such as Nemrut volcano. Instead, these new data display calc-alkaline to high-K calc-alkaline signatures broadly consistent with volcanic products from the CAVP, leaving open the possibility for future source attribution within this region.

## Technical Validation

### Accuracy and precision of glass shard compositions

**Fig. 4:**
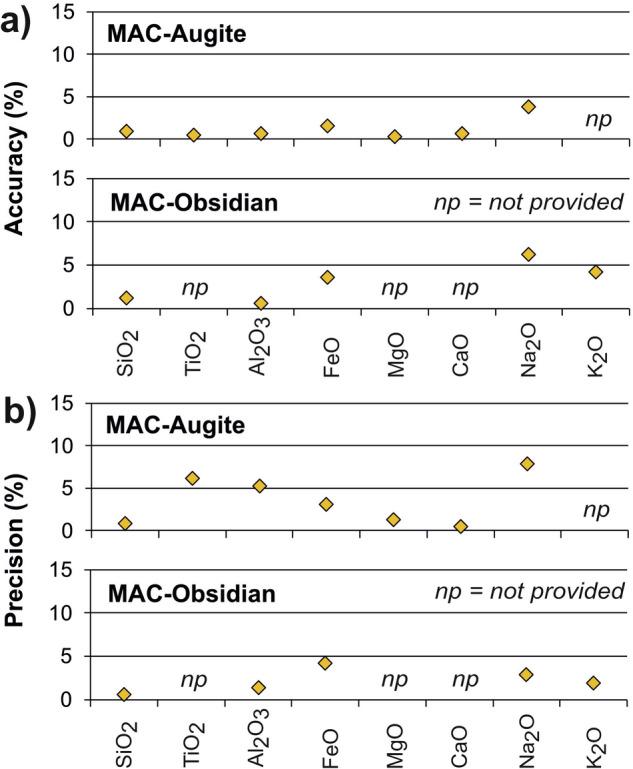
Accuracy (**a**) and precision (**b**) of major element analyses obtained by electron probe micro-analysis (EPMA), calculated using the secondary reference materials MAC-Augite and MAC-Obsidian. Accuracy and precision are generally better than 5% for most major oxides, apart from Na₂O in MAC-Obsidian, which reaches values close to 6%. Slightly higher precision values (5–8%) are observed for TiO₂, Al₂O₃, and Na₂O in MAC-Augite. Oxides labelled as “np” indicate elements not provided by the reference material.

For EPMA data, MAC-Obsidian and MAC-Augite show analytical accuracies better than 5% for all major oxides, with the only exception of Na_2_O in MAC-Obsidian, for which accuracy approaches 6% (Fig. 4a). Analytical precision is also generally better than 5%, except for TiO_2_, Al_2_O_3_ and Na_2_O in MAC-Augite, which range between 5% and 8% (Fig. 4b). These values compare favorably with accuracy and precision of EPMA analyses from Cullen *et al*.^3^, as determined using the Icelandic obsidian reference material ATHO-G (MPI-DING glass from the Max Planck Institute for Chemistry)^25^. Based on replicate analyses of ATHO-G reported by the authors, analytical accuracy is better than 4% for all major oxides, except for MgO and Na_2_O, which yield values of 11% and 10%, respectively. Analytical precision is generally better than 3%, except for MgO and TiO_2_, which exhibit values of 21% and 11%, respectively. For a rigorous statistical comparison of the dataset from this study with that of Cullen *et al*.^3^, data dispersion for each analyzed sample was first quantified using the mean, standard deviation, and coefficient of variation for all major oxides. Subsequently, hypothesis-testing criteria were applied using formal distribution overlap metrics, whereby 95% confidence intervals of the means were computed and the fraction of shared value ranges between datasets was evaluated. By propagating the analytical uncertainties of EPMA measurements across all major oxides in different samples, the resulting overlap coefficient ranges from 50% to 77%, indicating a statistically meaningful correspondence between datasets while accounting for differences in EPMA calibration, sample size, and the intrinsic variability of natural materials.

**Fig. 5:**
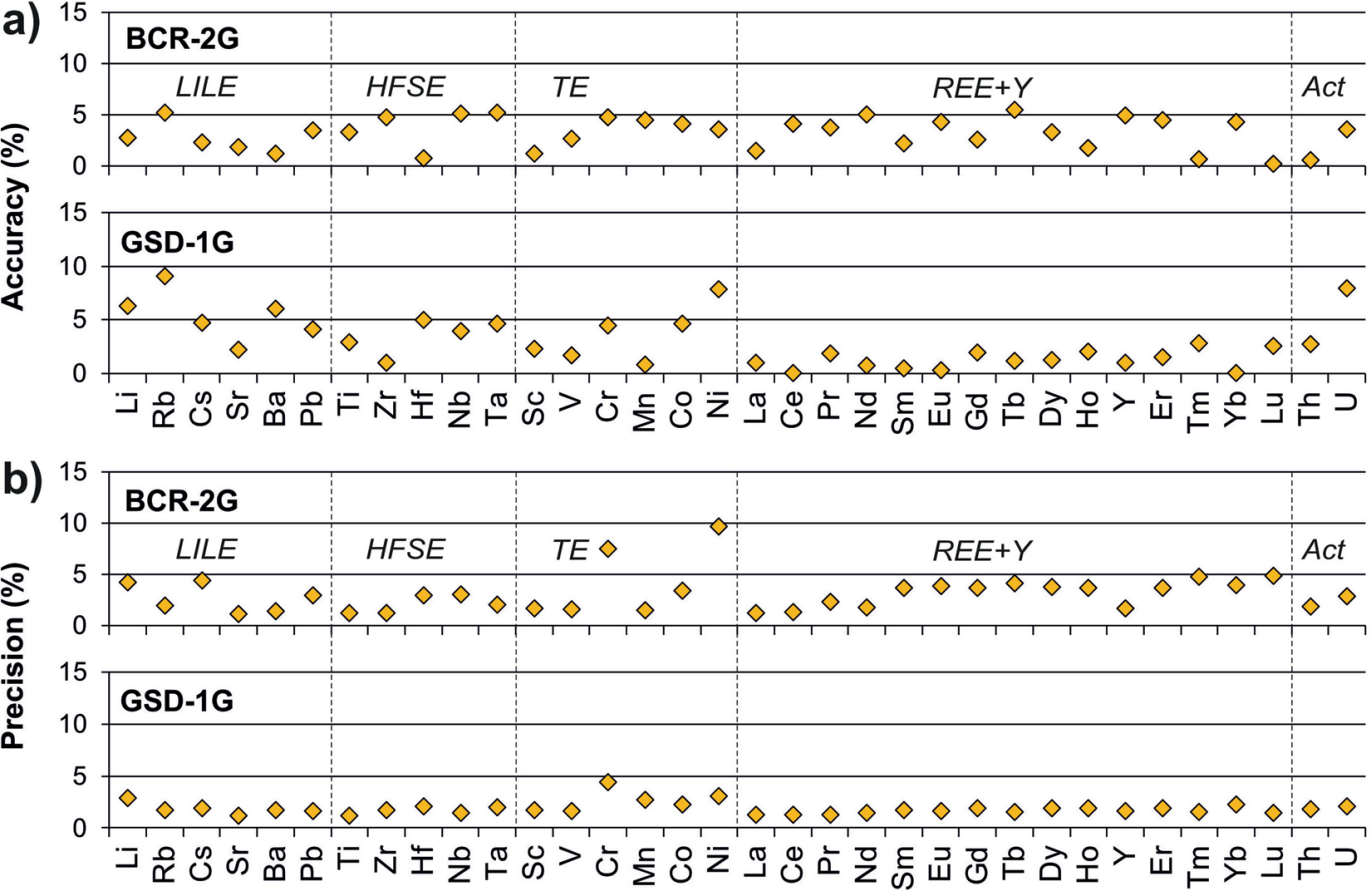
Accuracy (**a**) and precision (**b**) of trace element analyses obtained by sector-field laser ablation ICP-MS, calculated using the USGS reference materials BCR-2G and GSD-1G and grouped by geochemical affinity (large ion lithophile elements, high field strength elements, transition elements, rare earth elements + Y, and actinides). Accuracy is generally better than 6% for most elements, with slightly higher deviations (up to ~8–9%) for selected elements in GSD-1G. Precision is typically better than 5%, with higher values (7–10%) observed for Cr and Ni in BCR-2G.

For the LA-ICP-MS analyses, a limited number of trace elements belonging to the LILE (Rb), TE (Ni), and Act (U) in GSD-1G show slightly lower accuracies, in the range of 8-9% (Fig. 5a). Precision is typically better than 5%, except for a few TE (Cr, Ni) in BCR-2G, which exhibit higher values between 7% and 10% (Fig. 5b). Major and trace element data for the secondary reference materials are available for download from the Figshare repository^23^ (10.6084/m9.figshare.31223815).

### Statistically based petrochemical validation

**Fig. 6:**
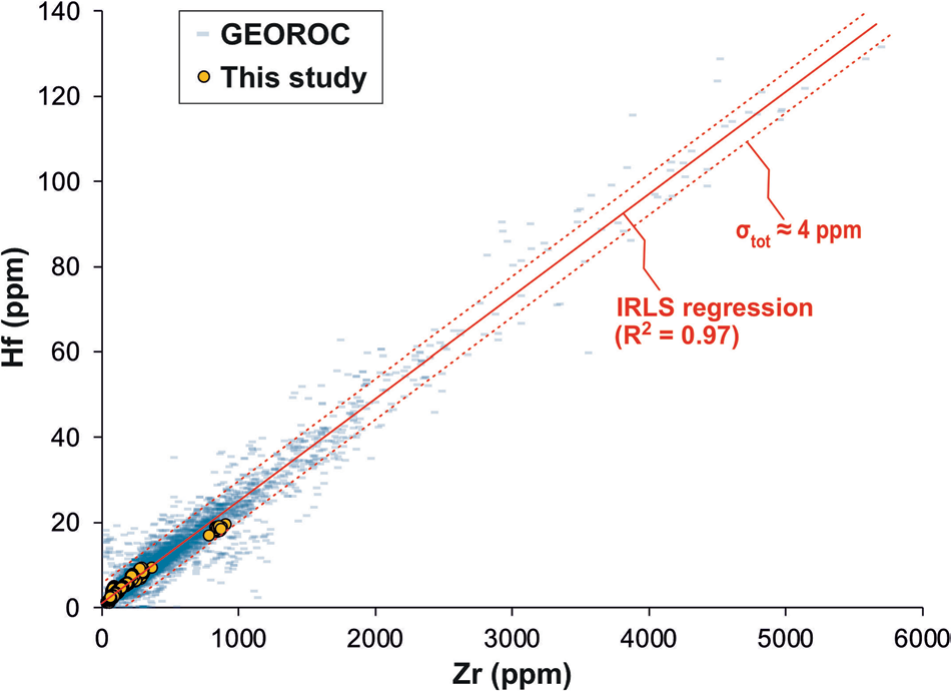
Petrochemical validation of the glass shard dataset from this study based on Zr and Hf concentrations. Glass shard compositions are compared with natural rhyolitic rocks from the GEOROC database (https://georoc.eu/). Iteratively reweighted least-squares (IRLS) regression of the rock data yields a high coefficient of determination (*R*^*2*^ = 0.97). The variance of the glass shard dataset is consistent with a total standard error of ∼4 ppm, reflecting both the intrinsic scatter of the IRLS regression and the natural variability of the population.

To assess the robustness and geochemical consistency of the dataset presented in this study, Zr and Hf concentrations in volcanic glass shards are compared in Fig. 6 with those of rhyolitic rocks compiled in the GEOROC database (https://georoc.eu/). Zr and Hf were selected because they belong to the same HFSE group and exhibit nearly identical, highly incompatible behavior during magma crystallization and differentiation, resulting in well-defined and internally consistent geochemical systematics. To retain the full statistical structure of the GEOROC database, we performed a robust linear regression using an iteratively reweighted least-squares (IRLS) method based on Huber weighting^26,27^. This approach progressively down-weights data points with large residuals while preserving high leverage for statistically frequent observations. The IRLS regression procedure was implemented in a custom Excel spreadsheet available from the Figshare repository to ensure full transparency and reproducibility of the statistical validation. An initial ordinary least squares (OLS) regression was first computed to obtain starting estimates of the slope and intercept of the linear model:

1 $$Y={aX}+b$$

where *Y* and *X* refer to Hf and Zr concentrations, respectively. Using these initial coefficients, fitted values ${\hat{Y}}_{i}$ were calculated for each observation, and regression residuals ${r}_{i}$ were defined as:

2 $${r}_{i}={Y}_{i}-{\hat{Y}}_{i}$$

The dispersion of the residuals was then estimated using a robust scale estimator *σ* based on the median absolute deviation (MAD)^28^:

3 $$\sigma =\frac{{med}\left(|{r}_{i}|\right)}{k}$$

where ${med}(\bullet )$ is the mean operator and *k* = 0.6745 is the normal-consistency constant, which corresponds to the median of the absolute value of a standard normal random variable and ensures consistency of the median absolute deviation with the standard deviation for normally distributed residuals. The estimator *σ* provides a robust measure of variability that is minimally affected by extreme values and remains consistent for normally distributed residuals. Data weights were assigned using the Huber weighting function^26,29^, which applies full weight to observations with small residuals and progressively reduces the influence of points with large deviations:

4 $${\omega }_{i}=\left\{\begin{array}{c}1,\,|{r}_{i}|\le c\sigma \\ \frac{c\sigma }{|{r}_{i}|},\,|{r}_{i}| > c\sigma \end{array}\right.$$

where *c* = 1.345 is the standard Huber tuning constant, chosen to ensure approximately 95% asymptotic efficiency relative to OLS for Gaussian residuals.

The weighted regression was implemented by transforming the original variables according to:

5 $${X}_{i,\omega }=\sqrt{{\omega }_{i}}{X}_{i}$$

and

6 $${Y}_{i,\omega }=\sqrt{{\omega }_{i}}{Y}_{i}$$

The regression coefficients were recomputed using weighted least squares on the transformed variables. This formulation is mathematically equivalent to solving the IRLS normal equations^27^.

The procedure was iterated through five loops, each involving recalculation of residuals, updating of the robust scale estimate and weights, and refitting of the weighted regression, until convergence was achieved. Convergence was defined by negligible changes in the estimated slope and intercept between successive iterations. In practice, stable solutions were typically reached within three to five iterations, consistent with previous applications of IRLS methods^30^. A formal ${z}_{i}$-based filtering criterion was applied for data not representative of natural geochemical variability, where ${z}_{i}$ denotes the standardized residual. This quantity, equivalent to *z*-score for normally distributed errors and commonly known as a studentized residual in regression analyses, is defined as:

7 $${z}_{i}=\frac{{r}_{i}}{\sigma }$$

Standardized residuals exceeding $|{z}_{i}|$ = 3 were examined as potential outliers, whereas extreme values with $|{z}_{i}|$ > 20 were identified as artefacts that compromise regression robustness and were therefore excluded from the statistical analysis.

The resulting IRLS regression equation exhibits the following form (Fig. 6):

8 $${Hf}({ppm})=0.02407\left({\rm{\pm }}0.00004\right)\times {Zr}({ppm})+1.04127\left(\pm 0.01179\right)$$

The coefficient of determination is high (*R*^*2*^ = 0.97), indicating that the IRLS model successfully reproduces 97% of the variance in the data and supporting the robustness of the regression fit (Fig. 6). Both the root mean squared error (*RMSE*), and the standard error (*SE*) of the estimate are relatively low, with *RMSE* = 0.92 ppm and *SE* = 0.92 ppm, further indicating a good overall fit of the IRLS model (Fig. 6).

For data representing a natural population, such as concentrations of trace elements in glasses and rocks, the overall natural variability is quantified using the standard deviation of multiple similar samples:

9 $${\sigma }_{{data}}=\sqrt{\frac{{\sum }_{i=1}^{N}{({X}_{i}-{\bar{X}}_{i})}^{2}}{N-1}}$$

where *X*_*i*_ is the value of the *i*-th sample, ${\bar{X}}_{i}$ is the mean of all samples, and *N* is the total number of samples. Once *σ*_*data*_ is determined, it is combined with the standard error of estimate of the IRLS regression *σ*_*SE*_ to obtain the total uncertainty *σ*_*tot*_:

10 $${\sigma }_{{tot}}=\sqrt{{\sigma }_{{SE}}^{2}+{\sigma }_{{data}}^{2}}$$

The calculated total uncertainty of ∼4 ppm reflects the combined contributions of the intrinsic scatter of the IRLS model and the variability inherent to the natural population. Agreement of the dataset presented here with the natural population was quantified by normalizing the residuals *r*_*i*_ by *σ*_*tot*_ to obtain *z*-scores. Values with $\left|\left.{z}_{i}\right|\right.$ ≤ 4 correspond to deviations within *σ*_*tot*_ and are considered consistent with the regression. In contrast, values with $\left|\left.{z}_{i}\right|\right.$ > 4 exceed the expected uncertainty of the population data. This approach provides a straightforward and quantitative criterion to assess whether independent measurements conform to the established natural variability of glasses and rocks. All data reported in this study satisfy the $\left|\left.{z}_{i}\right|\right.$ ≤ 4 thresholds, indicating consistency with the expected confidence interval for natural geochemical datasets (Fig. 6).

In summary, the statistically based petrochemical validation shows that the Zr-Hf systematics of the analyzed glass shards are consistent with the expected behavior of these highly incompatible elements in rhyolitic compositions. The IRLS regression provides an independent benchmark against which the internal geochemical coherence of the dataset can be evaluated, while the adopted uncertainty envelope accounts for both regression scatter and natural compositional variability. The fact that all analyzed glass shard data fall within this uncertainty criterion supports the robustness of the dataset and confirms its suitability for future comparative tephrochronological and geochemical correlation studies.

## Usage Notes

The full dataset, including the geochemical spreadsheets, representative microscopy images, and calculation workflows, is available via the Figshare repository described in the Data Records section^23^.

## Data Availability

No custom code has been used.
